# Pituitary Apoplexy Following Gonadotropin-Releasing Hormone Agonist Therapy: A Rare and Life-Threatening Complication

**DOI:** 10.7759/cureus.83531

**Published:** 2025-05-05

**Authors:** Aytan Naghiyeva, Nadia Smati, Calixto-Hope G Lucas, Brandi R Page, Mihail Zilbermint

**Affiliations:** 1 Department of Endocrinology, Diabetes, and Metabolism, Ege Hospital, Baku, AZE; 2 Department of Internal Medicine, Kaiser Permanente, Gaithersburg, USA; 3 Department of Pathology, Johns Hopkins University School of Medicine, Baltimore, USA; 4 Department of Head and Neck Radiation Oncology, Johns Hopkins University School of Medicine, Baltimore, USA; 5 Department of Radiation Oncology, Suburban Hospital, Bethesda, USA; 6 Division of Hospital Medicine, Johns Hopkins Community Physicians, Baltimore, USA; 7 Division of Endocrinology, Diabetes, and Metabolism, Johns Hopkins University School of Medicine, Baltimore, USA; 8 Division of Endocrinology, Diabetes, and Metabolism, Suburban Hospital, Bethesda, USA

**Keywords:** gnrh agonist, gonadotroph adenoma, gonadotropin-releasing hormone (gnrh), leuprolide, pituitary apoplexy, transsphenoidal pituitary surgery

## Abstract

Pituitary apoplexy is a rare but potentially life-threatening endocrine emergency characterized by acute hemorrhage or infarction of the pituitary gland. While often associated with pre-existing adenomas, it may also occur in previously normal glands. Risk factors include surgery, pregnancy, and medications such as gonadotropin-releasing hormone (GnRH) agonists, which are rarely implicated in pituitary apoplexy.

We present the case of a 59-year-old male with a history of type 2 diabetes mellitus, hypertension, hyperlipidemia, and prostate cancer who developed intractable headache, nausea, vomiting, photophobia, and blurred vision shortly after receiving a leuprolide, GnRH agonist injection, for the treatment of prostate cancer. Brain imaging revealed a sellar and suprasellar pituitary tumor with hemorrhage. He was treated with intravenous fluids and steroids. The patient underwent emergent transsphenoidal hypophysectomy surgery, and pathology confirmed a gonadotroph adenoma with necrosis. The patient was discharged in stable condition.

Pituitary apoplexy following GnRH agonist therapy is exceedingly rare, with fewer than 30 cases reported in the public domain. Proposed mechanisms include sudden cell shrinkage and metabolic hyperactivity within the pituitary gland. This case underscores the importance of recognizing the clinical presentation of pituitary apoplexy, as timely diagnosis and treatment are crucial to preventing severe neurological and endocrine sequelae.

Clinicians should maintain a high index of suspicion for pituitary apoplexy in patients presenting with acute neurological or endocrine symptoms following GnRH agonist therapy. Increased awareness and vigilance in at-risk populations are essential, as routine pre-screening for pituitary adenomas is not currently standard practice.

## Introduction

Pituitary apoplexy is a rare but potentially life-threatening endocrine emergency characterized by the sudden onset of hemorrhage or infarction within the pituitary gland [1]. This condition most commonly arises in the context of a pre-existing pituitary adenoma, although it can also occur in a previously normal gland [2]. While the exact etiology remains unclear in some cases, several well-established risk factors have been identified, including major surgery, pregnancy, stereotactic radiosurgery, anticoagulant therapy, coagulopathies associated with liver failure, and the administration of certain medications such as thyrotropin-releasing hormone, gonadotropin-releasing hormone (GnRH) agonists, bromocriptine, and cabergoline [3]. Recognizing these risk factors and the underlying pathophysiology is critical for prompt diagnosis and management, as pituitary apoplexy can have significant neurological and endocrinological sequelae if not treated appropriately [3].

The clinical presentation of pituitary apoplexy typically evolves over 24 to 48 hours, with severe headache and ocular palsies or visual field defects being hallmark features [4]. Additional manifestations may include cardiovascular collapse, altered consciousness, neck stiffness, and, in some cases, hypoglycemia. Rarely, bilateral cerebral infarction may occur, further complicating the clinical picture. Acute adrenal insufficiency is a frequent and critical consequence due to the abrupt loss of adrenocorticotropic hormone, which may be exacerbated by disordered intravascular coagulation, heparin administration, or concurrent central nervous system hemorrhage [5]. Imaging, particularly magnetic resonance imaging (MRI), is pivotal in diagnosis [6]. Typical findings include intrapituitary or intra-adenoma hemorrhage, stalk deviation, compression of normal pituitary tissue, and in severe cases, evidence of parasellar hemorrhage [7].

## Case presentation

A 59-year-old male with a medical history of type 2 diabetes mellitus, hypertension, hyperlipidemia, and prostate cancer presented to the hospital with an intractable headache, nausea, vomiting, photophobia, and blurred vision. Symptoms began abruptly 15 minutes after receiving the first leuprolide injection for prostate cancer at his doctor’s office. On physical examination performed in the hospital, the patient’s vital signs were notable for a blood pressure of 146/94 mmHg and a pulse rate of 91 bpm. Visual fields were grossly intact, although the patient reported blurry vision in the right eye when tested in isolation. Cranial nerve examination was unremarkable, and the motor examination demonstrated normal strength (5/5) in all extremities without sensory deficits. Despite being cooperative, the patient appeared in moderate distress and was unable to get comfortable due to a persistent headache. Initial serum hormone workup is summarized in Table 1.

**Table 1:** Laboratory test results

Test	Value	Reference range
Serum cortisol (random)	3.7 mcg/dL	4.6–23.4 mcg/dL
Thyroid-stimulating hormone	0.5 mcIU/mL	0.50–4.50 mcIU/mL
Free thyroxine	2.1 ng/dL	0.9–1.7 ng/dL
Luteinizing hormone	3.8 mIU/mL	1.7–11.2 mIU/mL
Follicle-stimulating hormone	12.3 mIU/mL	1.5–12.4 mIU/mL
Prolactin	4.3 ng/mL	3-13 ng/mL
Total testosterone	340.0 ng/dL	264–916 ng/dL
Adrenocorticotropic hormone	16 pg/mL	6–50 pg/mL
Insulin-like growth factor-1	303 ng/mL	50–317 ng/mL

**Figure 1:**
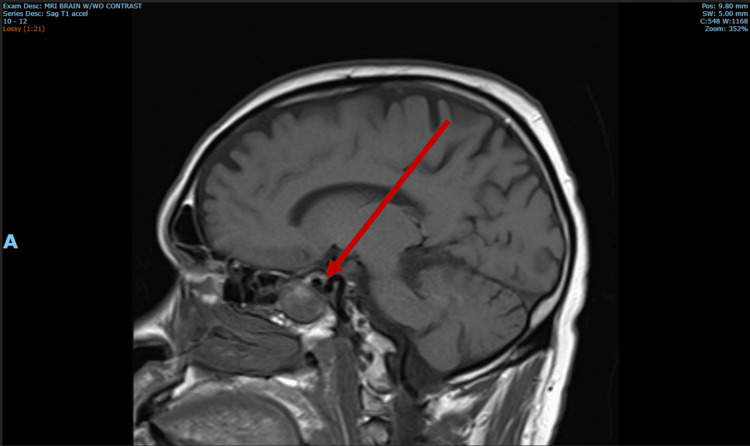
Magnetic resonance imaging of the brain. Magnetic resonance imaging of the brain revealed a large heterogeneous enhancing mass expanding the sella, extending inferiorly into and expanding the left sphenoid sinus, and extending superiorly into the suprasellar cistern causing mild mass effect on the optic chiasm, measuring 2.8 x 3.4 x 3.4 cm. There was no definite cavernous sinus invasion. There was mild patchy intrinsic T1 hyperintensity throughout the mass, with small central areas of susceptibility artifact particularly within the left sphenoid sinus.

The magnetic resonance imaging of the brain demonstrated a large sellar and suprasellar pituitary tumor measuring 2.8 x 3.4 x 3.4 cm with mild compression of the optic chiasm, T1 hyperintensity, and evidence of hemorrhage (Figure 1).

Brief hospital course

**Figure 2:**
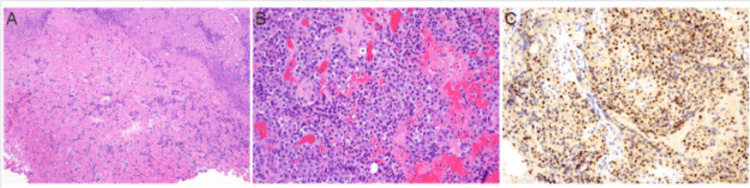
Transsphenoidal resection of pituitary tissue A. Hematoxylin- and eosin-stained sections revealed large sheets of necrotic debris with mixed inflammation (100x magnification). B. Small areas of viable adenoma were also seen, composed of monomorphic cells with fine chromatin and granular eosinophilic cytoplasm arranged in sheets (200x magnification). C. The adenoma cells were diffusely immunoreactive for steroidogenic factor 1 (200x magnification) while negative for other pituitary transcription factors and hormone stains.

The patient was administered intravenous steroids (loading dose of 16 mg of dexamethasone and 4 mg every six hours thereafter), and neurosurgery was emergently consulted. The patient underwent transsphenoidal pituitary surgery due to the compression of optic apparatus and vision changes. The pathology revealed large sheets of necrotic debris with mixed inflammation and small areas of viable adenoma, composed of monomorphic cells with fine chromatin and granular eosinophilic cytoplasm (Figure 2). The pathology of the tumor was consistent with gonadotroph adenoma with necrosis. The patient was discharged from the hospital in stable condition.

Clinical challenge

The primary clinical challenge in this case lies in the timely identification of pituitary apoplexy as the cause of the patient's acute symptoms given the nonspecific nature of the presentation and the potential for misdiagnosis as a more common condition, such as medication side effects or metastatic disease. The rare association between GnRH agonist therapy and pituitary hemorrhage adds an additional layer of complexity to the diagnosis.

## Discussion

Pituitary apoplexy is a rare medical emergency characterized by acute hemorrhage or infarction of the pituitary gland, most commonly occurring in the context of pre-existing adenomas, with an estimated prevalence of 6.2 cases per 100,000 people [8]. This condition can result in severe neurological and endocrine complications, including the sudden onset of headache, visual disturbances, and hormonal deficiencies. This condition can lead to a cascade of severe neurological and endocrine complications, such as the abrupt onset of debilitating headaches, dramatic visual disturbances such as tunnel vision or even blindness, and a rapid depletion of critical hormones that can push the body into life-threatening adrenal crises or other endocrine emergencies. Each symptom not only signals the gravity of the condition but also underscores the intricate interplay between the gland's anatomical location and its pivotal hormonal functions [9].

Notably, pituitary apoplexy has been rarely associated with the use of GnRH agonists. This class of medications is widely used for treating hormone-responsive conditions such as prostate cancer, endometriosis, uterine leiomyomas, and breast cancer. Additionally, GnRH agonists have several off-label applications, including use in transgender therapy, in vitro fertilization, premenstrual dysphoric disorder, and central precocious puberty [10]. Since this complication was first reported in 1995, fewer than 30 cases have been documented. The onset of symptoms following GnRH agonist administration has shown significant variability, ranging from minutes after injection to as long as six months after the last dose [11]. A case series revealed that, similar to our patient, the majority of reported cases did not have a previously known pituitary tumor [12].

The exact mechanism by which leuprolide induces pituitary apoplexy remains unclear. Guerra et al. suggested a biphasic process, proposing that pituitary apoplexy induced by GnRH agonists may occur in two phases: acute and subacute [13]. This dual pathophysiology model helps explain the varying features seen in reported cases, particularly in terms of symptom onset timing. In instances where symptoms developed within minutes or hours of drug administration (acute phase), a combination of cell degranulation, shrinkage, and increased metabolic activity in poorly perfused adenomatous pituitary tissue (due to an abnormal capillary network) could account for the event. In contrast, in patients whose symptoms appeared later (subacute phase), it was proposed that the stimulation of luteinizing hormone (LH) secretion, leading to cell growth and protein synthesis, could affect tumor size and increase intrasellar pressure, resulting in widespread ischemia and bleeding. Additionally, it has been suggested that gonadotroph adenomas are the most common tumors associated with PA. The binding of GnRH agonists to GnRH receptors on gonadotropin-secreting pituitary cells leads to a significant increase in LH and follicle-stimulating hormone (FSH) levels, and this hormonal stimulation might promote tumor growth, further exacerbating tissue infarction [13]. GnRH agonists initially stimulate the pituitary gland to release gonadotropins, such as FSH and LH, but prolonged exposure leads to downregulation of GnRH receptors and subsequent hypogonadism due to decreased sex hormone levels [14].

While pituitary apoplexy is a rare complication of leuprolide injection, it represents a potentially life-threatening medical emergency. Although this complication has been acknowledged in post-market surveillance by the U.S. Food and Drug Administration, it remains underrecognized in clinical practice [15]. Routine evaluations typically do not include pre-screening for pituitary adenomas before administering GnRH agonist injections despite the potential risk of complications such as pituitary apoplexy in predisposed individuals [16].

## Conclusions

Given the critical importance of early diagnosis and treatment in improving outcomes for pituitary apoplexy, it is essential for physicians to recognize this rare but potentially life-threatening complication when counseling patients. Clinicians should remain vigilant for signs and symptoms of pituitary apoplexy, particularly in patients with a history of GnRH agonist treatment. The absence of comprehensive clinical guidelines further highlights the need for healthcare professionals to maintain a high index of suspicion, especially in individuals undergoing GnRH agonist therapy or those with known pituitary lesions.
